# Protocol for the REVISIT-BPD Trial, a Randomized Controlled Trial Testing the Effectiveness of an Internet-Based Self-Management Intervention in the Treatment of Borderline Personality Disorder (BPD)

**DOI:** 10.3389/fpsyt.2018.00439

**Published:** 2018-09-21

**Authors:** Jan Philipp Klein, Andrea Hauer, Thomas Berger, Eva Fassbinder, Ulrich Schweiger, Gitta Jacob

**Affiliations:** ^1^Department of Psychiatry and Psychotherapy, Lübeck University, Lübeck, Germany; ^2^Gaia AG, Hamburg, Germany; ^3^Department of Clinical Psychology and Psychotherapy, Bern University, Bern, Switzerland

**Keywords:** psychotherapy, randomized controlled trial, internet, schema therapy, telemedicine

## Abstract

**Introduction:** Borderline Personality Disorder (BPD) is a prevalent condition that is often under-treated. This is partly because very few psychotherapists offer treatment for this disabling disorder. Internet-based self-management interventions could contribute to reducing the treatment gap but—mainly due to safety concerns—these have never been tested for BPD in controlled trials.

**Methods:** Patients with BPD will be recruited primarily via the internet and randomized to two groups: care as usual (CAU) alone) or the self-management intervention *priovi*® in addition to CAU. At the end of the diagnostic interview, all participants will discuss an emergency plan. The main outcome measure is the clinician-rated symptom severity using the BPD Severity Index (BPDSI). Secondary outcome measures include a range of self-reported scales, an SCID-diagnosis of BPD and several safety parameters including serious adverse events (e.g., a life-threatening event, hospitalization or suicide attempt).

**Discussion:** This trial will evaluate the effectiveness of the self-management intervention, *priovi*, in reducing symptoms of BPD. It will also assess the safety of its use in this target population. If successful, this intervention would be the first comprehensive internet intervention for the treatment of BPD and complement the wide range of internet interventions effective in treating other mental disorders, particularly depression and anxiety disorders.

**Trial Registration:** NCT03418142 (clinicaltrials.gov) on January 23rd 2018.

**Trial status:** recruiting, currently *N* = 108 (July 2018).

## Introduction

Borderline Personality Disorder (BPD) is a common mental disorder characterized by a pervasive pattern of instability in interpersonal relationships, self-image and affect as well as marked display of impulsivity (1). The prevalence of BPD is about 2% with higher prevalence rates in women than in men (2, 3). In Germany, the annual treatment costs for BPD amount to about 15% of the total costs incurred for mental disorders (4). About 75% of these costs are due to inpatient treatment, which can be substantially reduced by effective treatment (5). Although a number of effective psychological treatments exist for BPD (6), only about one-in-four patients receive psychotherapy even in relatively well-developed health care systems (7). Reasons for this treatment gap include unavailability of trained psychotherapists. In one study involving over 150 psychotherapists, about one-in-five outrightly refused to treat patients with BPD “out of principle” (8).

Internet interventions are considered a viable option to help reduce the treatment gap in mental health (9). Broadly, these interventions can be divided into teletherapy interventions and self-management interventions (10). In *teletherapy interventions*, therapists communicate with their patients via the internet (e.g., e-mail or video chat). In *self-management interventions*, knowledge and techniques based on evidence-based psychotherapeutic methods are taught by an internet-based computer program instead of a therapist. Mostly based on the principles of cognitive behavioral therapy, these interventions can be accessed on internet-enabled desktop computers as well as mobile devices. Self-management interventions can be offered in *guided* or *unguided* form. Guidance often comprises electronic messages that are aimed at increasing adherence to the intervention.

In general, self-management interventions have a number of advantages. These include scalability and availability that is independent of the location and opening hours of a therapist. They may thus be an alternative for patients with mental illness who do not or cannot seek therapy either because a therapist is not available where they live (e.g., patients living in rural or remote areas) or because they are unable to find some free time during office hours of most therapists (e.g., patients who work and raise children) (11). In the treatment of BPD, self-management interventions can have the additional advantage of being available in times of crises when a therapist is not immediately available. Here, they can offer *in vivo* skills coaching (12) and thus facilitate the transfer or adoption of newly acquired skills in everyday life (13).

Disadvantages of self-management interventions include the fact that there are a number of interventions on the market that have never been rigorously tested in clinical trials (10). Also, while randomized trials have found no difference between face-to-face psychotherapy and guided self-management interventions in terms of dropout and effect sizes (14, 15), these outcomes have been found to be less favorable in purely unguided self-management interventions (16, 17). An additional disadvantage of self-management interventions is the fact that they can only react to acute crises in a very limited fashion (usually by referring to emergency psychiatric services). As will be discussed below, this limitation is of particular concern in the treatment of patients with BPD.

Internet interventions have been successfully tested in many randomized clinical trials (RCT) that have been summarized in numerous meta-analyses. These meta-analyses attest to the efficacy and effectiveness of these interventions in treating symptoms of depression (18) and anxiety disorders (19, 20). These interventions have also been used successfully in RCTs of patients with post-traumatic stress disorder (21), substance use disorder (22), and even schizophrenia (23). To our knowledge, there is no randomized trial of a self-management intervention in the treatment of BPD.

Self-management interventions for BPD have been tested in open-label pilot studies. However, in these studies, the interventions have been applied in a *blended format*, i.e., the intervention was used in addition to face-to-face psychotherapy. These interventions include the “DBT Coach” that can be used as an adjunct to Dialectic Behavioral Therapy (DBT) (12, 24) and “priovi®,” a program that is based on the principles of Schema Therapy (13). Offered in the traditional face-to-face format, both DBT and Schema Therapy have been shown to be effective in the treatment of BPD (25). The pilot studies on the aforementioned self-management interventions have concluded that they are well accepted by patients and can contribute to the reduction of symptoms of BPD. There is also an ongoing randomized study comparing an internet-delivered Dialectical Behavioral Therapy Skills Training (iDBT-ST) to a waitlist control condition in suicidal individuals engaging in heavy episodic drinking (26).

Experts in the treatment of BPD agree that self-management interventions should be used mainly in the blended format described above. Reasons for this preference include the opportunity for corrective experiences with respect to dealing with conflict and intense emotions in face-to-face psychotherapy. Another reason against using self-management interventions as a stand-alone therapy for BPD are safety concerns in a patient population that frequently engages in self-harm, suicide attempts, or other risky behaviors (13). On the other hand, a considerable proportion of individuals with untreated mental illness state that they prefer not to depend on a professional for the management of their disorder (11). Also, the aforementioned studies did not indicate possible harm that came from the use of self-management interventions in the treatment of BPD. A meta-analysis of studies with patients suffering from depressive symptoms found that compared with control conditions, harmful effects were less likely during the use of self-management interventions (27).

We have therefore decided to conduct the first randomized controlled trial of a self-management intervention based on Schema Therapy (priovi®) in the treatment of BPD. Given the characteristics of this patient population, particular emphasis has been placed on safety aspects during the design of this trial. This includes the discussion of a crisis plan with every participant of the trial and the careful assessment of negative effects and serious adverse events. We hypothesize that the use of this intervention in addition to care as usual (CAU) will be more effective than CAU alone in reducing symptoms of BPD over 1 year. We have chosen “care as usual” as the comparison condition because we consider it unethical to withhold treatment for 1 year in subjects suffering from a disorder that is as disabling and potentially dangerous as BPD. CAU was also chosen because it enhances the external validity of our findings by testing whether the intervention improves outcomes compared to the care usually received in the health care system (28). Additional analyses will concern the safety parameters and a broad range of secondary outcomes.

## Methods

In the following, we will describe the protocol for this trial entitled “REVISIT-BPD” (Research Evaluating the EffectiVeness of Adding an Internet-Based Self-Management Intervention to Usual Care in the Treatment of Borderline Personality Disorder). The trial is registered at ClinicalTrials.gov (NCT03418142). The description follows the SPIRIT guidelines (29) and the CONSORT guidelines for internet-based interventions (30).

### Trial design

This is a rater-blind, randomized (1:1), controlled, parallel group, superiority trial examining the effectiveness of an unguided internet-based self-management intervention in the treatment of BPD.

### Setting

The recruitment of study participants takes place via different channels. The most important recruitment channel is online (e.g., via Google ads and a dedicated Facebook page—https://www.facebook.com/priovistudie/). Internet users who enter certain search terms (e.g., borderline self-help, borderline study, priovi, etc.) on the Google search engine are shown a short ad that refers to the study homepage (https://priovi-studie.de/). Furthermore, the study will be advertised on patient platforms and via patient associations. In addition, we will recruit participants via health insurance companies; these will send out a letter about the study once to each insuree who is eligible to participate. Interested patients may also be referred to the study homepage via other means (e.g., a recommendation by their treating clinicians).

### Participants

Patients will be included in the study if they have a total score of at least 15 on the BPD Severity Index (BPDSI) (31, 32) and a diagnosis of BPD according to DSM-5 (at least five definite criteria) (1) as assessed by the structured clinical interview for DSM-IV (SCID) (33, 34). A BPDSI score of below 15 has empirically been shown to be the cutoff for recovery of BPD (31). Patients can also be included, if they only meet the criteria for a probable BPD diagnosis (three definite and at least two probable of the DSM-5 criteria), but only if they have already received a diagnosis of BPD by their treating clinician. All patients also have to be at least 18 years of age; provide informed consent; have an adequate command of the German language; consult a psychiatrist/psychotherapist before randomization for confirmation of their diagnosis; be willing and able to participate in the planned diagnostic assessments. They will be excluded from participating in the trial if they meet diagnostic criteria for any of the following diagnoses on the MINI diagnostic interview (35): psychotic disorder (patients will not be excluded if they experience only transient, stress-related paranoid ideation that is consistent with a diagnosis of BPD); primary diagnosis of substance use disorder; schizotypal disorder.

### Interventions

All participants are permitted to use any form of treatment, including psychotropic medication and psychotherapy. Following a pragmatic design approach, CAU is not influenced by the investigators (28). Participants in the control condition receive only CAU. In addition, they receive information regarding freely available self-help material on the internet, and they are offered access to the internet intervention after the last follow-up assessment.

Participants in the intervention group receive access to the unguided self-management intervention *priovi* in addition to CAU. They will be informed that priovi does not replace their ongoing psychotherapy but is merely an addition to it. The priovi intervention was developed and is owned by GAIA group, Hamburg, Germany. It is based on the principles of Schema Therapy. In Schema Therapy, problematic BPD behaviors and symptoms are connected with so-called schema modes, i.e., emotional states related to dysfunctional schemas such as mistrust/abuse or abandonment. Examples of typical schema modes in BPD patients include the *angry/impulsive child* mode (related to anger outbursts and impulsive behaviors) and the *detached protector* mode (related to problematic emotion-avoidance strategies such as dissociation). In Schema Therapy patients learn to identify their schema modes and to understand the biographical background of these modes. Further along, the treatment goals in Schema Therapy are also related to these modes and include helping the angry child mode find adequate ways to deal with anger or reassuring the detached protector mode so that patients can reduce their emotional avoidance and learn healthier strategies to deal with emotions and relationships.

Priovi is comprised of eight modules that are organized in simulated dialogues and can be accessed over a period of 1 year. These modules are divided in a psychoeducation phase (BPD symptoms, human needs, childhood abuse, and modes) and an intervention and exercise phase (these are tailored to the modes of the user and include cognitive techniques, imagery exercises, and affirmative audios). Each session is tailored to the current mood state of the user and thus addresses the frequent mood shifts associated with BPD. It is recommended to use the intervention twice a week for half an hour. Participants using the intervention on this regular basis can complete the full content in about 6 months, but it is recommended to use the intervention for the full year to continue with the exercises. A full description of the intervention can be found elsewhere (13).

The intervention will be unguided but participants who have not logged onto the intervention until the fifth day will receive a reminder that will once again explain the login procedure. Also, the intervention will send automated messages that are designed to motivate the user to engage with the intervention and the content covered in the intervention on a regular basis. Participants will also be able to contact a hotline to receive technical support. The hotline will refer to the treating psychiatrist / psychotherapist for management of acute crises.

### Sample size

The sample size calculation is based on the expected difference between the intervention and the control group in the severity of BPD (measured with the BPDSI) at the 12 months assessment. Based on an estimated effect size of Cohen's *d* = 0.40, a power of 0.80 and an alpha level of 0.05, 100 participants are required in each condition resulting in a target sample size of *N* = 200. The effect size estimate was based on a recent meta-analysis (6), where the between-group effect for add-on designs (in which both groups received CAU and one group received an additional BPD therapy) was *g* = 0.40.

### Procedure and assessments

After providing online informed consent, all prospective participants will be assessed via an online questionnaire and a telephone interview to establish if the inclusion criteria have been met. The assessment by online questionnaire and telephone interview will also be used to establish the baseline (T0). At the end of the telephone interview an individual crisis plan is discussed with all participants, i.e., whether they have contact information at hand of people they could contact in case of an acute crisis, e.g., acute suicidal ideation or impeding severe self-injury. Crisis contacts include both professionals (psychiatrists/psychotherapists) and friends. All participants will also be made aware of the nationwide emergency number−112.

Following the telephone interview, eligible participants will have to ask a psychiatrist or psychotherapist to confirm in writing (using a form supplied by the study staff) that they are suitable for participating in an RCT of a self-management intervention for BPD. After the receipt of this confirmation by a psychiatrist/psychotherapist, participants will then be randomized. They are contacted again after 3 months (T1), 6 months (T2), 9 months (T3), and 12 months (T4). All assessments include an online questionnaire and a telephone interview except for the T3 assessment that only comprises an online assessment. All assessments and the respective measures to be used can be found in Table 1.

**Table 1:** Schematic diagram of study timeline.

	**Study period**					
	**Enrolment**	**Allocation**	**Post-allocation**			
Timepoint	T0		*T1*	*T2*	*T3*	*T4*
**ENROLMENT**						
Informed consent	X					
Eligibility screen	X					
Psychiatrist /Psychotherapist Confirmation	X					
Allocation		X				
**INTERVENTIONS**						
Self-management intervention priovi® plus CAU			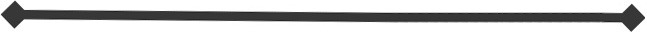			
CAU alone			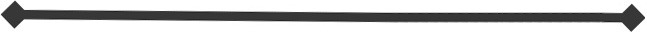			
**ASSESSMENTS**						
Borderline personality disorder severity index (BPDSI)	X		X	X		X
Structured clinical interview for DSM-IV (SCID)	X		X	X		X
Adverse or serious adverse events	X		X	X		X
Borderline personality disorder checklist	X		X	X	X	X
9-item Patient Health Questionnaire (PHQ-9)	X		X	X	X	X
7-item anxiety scale (GAD-7)	X		X	X	X	X
Quality of life questionnaire (EQ-5D-3L)	X		X	X	X	X
Negative effects questionnaire (NEQ)			X	X	X	X
Self-reported uncontrolled internet use	X		X	X	X	X

*CAU, care as usual; T0, baseline; T1, 3 months; T2, 6 months; T3, 9 months; T4, 12 months*.

### Randomization

Participants are randomized equally (1:1) into two groups (intervention or control). Randomization is stratified by the presence of a diagnosis of BPD (probable vs. definite diagnosis of BPD). Block randomization with variable block sizes is used. The allocation schedule was created by an independent investigator with a computerized random number generator; the other investigators are blind to this schedule. The allocation sequence is concealed from participants and all trial staff. Only the unblinded trial staff is informed about the randomization outcome. The unblinded trial staff will not be included in data collection or evaluation and is instructed not to inform the raters about the randomization outcomes. Additionally, all participants are informed three times (at the end of the first interview, via the mail invitation for the second interview and at the beginning of the second interview) not to disclose their group assignment to their interviewer and to reserve questions about the usage of the intervention program exclusively for priovi support or the study team.

### Outcome measures

All outcome measures are used in their German version. The main outcome measure is the Borderline Personality Disorder Severity Index (BPDSI) (31, 32), a clinician-rated semi-structured interview that is based on the DSM-IV criteria for BPD. It assesses the frequency of BPD-manifestations during the past 3 months. It has excellent psychometric properties: Cronbach's α = 0.85 in patients with BPD and interrater reliability of 0.98. It also has excellent criterion-validity and change sensitivity. All raters will have a bachelor or master degree in psychology, have experience in psychological diagnostic assessments and will have participated in a rater training. Before they are permitted to rate trial participants, raters will have to demonstrate adequate interrater reliability on an audiotaped interview. For each rater, we will also assess the interrater reliability of their third interview.

Secondary study outcomes and safety parameters that will also be assessed during the diagnostic interview include the presence of a diagnosis of BPD on the structured clinical interview for DSM-IV (SCID) (33, 34) and the presence of adverse or serious adverse events. The assessment of adverse events follows recommendations established in pharmacological research (36). Using a semi-structured interview, participants are asked whether they have experienced any of the following events during the observation period (past 33 months for baseline and since last assessment for all other assessments): life-threatening events (e.g., self-injury, drug intoxication, accidents, etc.), medical occurrences that require hospitalization and suicide attempts.

The following secondary outcomes and safety parameters are then assessed using self-report: severity of BPD using the BPD-Checklist (37), depressive symptoms using the 9-item Patient Health Questionnaire (PHQ-9) (38), symptoms of anxiety using a 7-item anxiety scale (GAD-7) (39), quality of life (QoL) using the five-dimension three-level version of the questionnaire developed by the EuroQoL group (EQ-5D-3L) (40) and negative effects of the intervention using the Negative Effects Questionnaire (NEQ) (41). Participants are also asked about the parallel use of psychotherapy.

As a further safety parameter, we will employ a newly developed scale assessing self-reported uncontrolled internet use. It is based on the Compulsive Internet Use Scale (42) but comprises only two items that assess the frequency of using the internet longer than intended and avoiding work or family obligations due to internet use.

### Statistical analysis

All continuous outcomes will be analyzed as a change from baseline with linear mixed models (LMM). These have the advantage of using all available data of each subject and they also offer the opportunity to choose an appropriate covariance structure reflecting the potential dependence due to repeated measurements (43). Adjustment for baseline measure will be chosen as this increases statistical power and accounts for regression to the mean (44). Missing values will be substituted using multiple imputations (with 50 imputations per missing value) to estimate missing scores by evaluating the relationships between observed and missing scores as well as baseline scores. For dichotomous outcomes, we will calculate logistic regression analyses that are adjusted for baseline values and time-to-event analyses (Kaplan–Meier survival analyses and Cox proportional hazards regression).

#### Primary analysis

The primary analysis for all the outcomes will be an LMM analysis on the intention-to-treat sample (ITT analysis), which includes all randomized participants. These analyses will include a random intercept for the participant and adjustment for baseline measure. The following will be entered as a fixed effect: time, study group, diagnosis of BPD, and the time X group interaction. A covariance structure will be chosen based on Akaike's Information Criterion (AIC) from a fixed set of candidate structures, namely a first order autoregressive (AR1), diagonal or scaled identity structure or heterogeneous versions thereof. The study hypothesis will be tested on the main effect for group. The “time x group” interaction will be reported to establish whether the between-group effect changes over the 1 year assessment period.

#### Sensitivity analysis

We will conduct sensitivity analyses to determine the robustness of our results for the main outcome (BPDSI). In an *adjusted analysis*, we will correct for the following baseline variables: age, sex as well as baseline severity and diagnosis of BPD. In a *per-protocol analysis*, we will include participants randomized to the intervention group only if they have used the intervention for at least 3 h and compare these to all participants in the CAU group.

#### Subgroup analyses

We will also perform subgroup analyses to establish whether certain subgroup variables moderate the effect of the intervention on the main outcome (BPDSI). The hypothesis of difference in treatment effects will be tested using the “group X subgroup variable” interaction. In one subgroup analysis, we will test the influence of a diagnosis of BPD (as assessed during the SCID screening interview). In another subgroup analysis, we will test the influence of concomitant psychotherapy (as self-reported by the participants).

#### Exploratory analyses

Further exploratory analyses will be conducted. One exploratory analysis will examine predictors and moderators of serious adverse events. Another analysis will deal with the adherence-outcome association as adherence has been shown to improve outcome in a self-management intervention for depression (45).

### Data management

The steering committee will perform internal audits to ascertain that this protocol is adhered to, all trial staff has the required qualifications and that the data quality is adequate (e.g., range checks for data values). During these audits, frequencies of serious adverse events will be compared between groups. The trial will be terminated prematurely if serious adverse events occur significantly more frequently in the intervention group. No independent Data Monitoring Committee has been established however and no interim analyses for the main outcome or other outcomes other than safety will be conducted.

## Discussion

The REVISIT-BPD trial will be the first RCT of a self-management intervention based on Schema Therapy in the treatment of BPD. It is designed to establish the effectiveness of this intervention in reducing clinician-rated symptoms of BPD. Various measures have been taken to ensure the safety of all participants in this trial and to monitor the occurrence of negative effects and serious adverse events.

Ours is among the few trials of psychological interventions that systematically assesses negative effects (41) and the first to also assess the occurrence of serious adverse events in clinical interviews. We will, therefore, ensure the safety of our trial participants and also be able to examine the relative frequency of serious adverse events in the intervention compared to the control group and possibly also establish predictors and moderators of serious adverse events in this population. Furthermore, this trial stands apart from most trials of self-management interventions in that the symptom course is assessed not only using self-report but also clinician-rated scales. Only very few RCTs of self-management interventions for depression have used clinician ratings (46, 47).

A possible weakness of our design is the fact that patients will also be included in this trial if they only partially meet DSM-5 criteria for BPD (three definite and at least two probable criteria) as long as they score at least 15 on the BPDSI and have previously been diagnosed with BPD by their psychiatrist or psychotherapist. We decided to include patients with such a probable diagnosis of BPD into the study because they already have a pre-existing diagnosis of BPD made by their treating clinician. Most likely, this diagnosis is not based on clinical interview but rather on a more long standing clinical evaluation of the patient and should therefore not be called into question if BPD symptoms are sufficiently severe. We have stratified the randomization by diagnosis of BPD (probable vs. definite) to assure a good balance of this characteristic across both groups.

A further limitation is the fact that we are not collecting detailed data on the use of concomitant therapy. In fact, we are only inquiring about parallel psychotherapies. In a previous, much larger trial of a self-management intervention for patients suffering from depressive symptoms, we found that the use of concomitant therapies did not differ between study groups over 1 year (48).

It should also be kept in mind that many studies of self-management interventions yielded smaller effect sizes than the effect we have based our sample size estimate on. The between-group effect for self-management interventions for depression can range from *g* = 0.27 when using unguided interventions in patients self-reporting depressive symptoms (18) to *g* = 0.90 in the treatment of patients with an established diagnosis of depression (49). We therefore think that our sample size estimate is realistic but it may prove to be too small. Consequently, our trial can theoretically result in a positive result with a smaller than expected between-group effect and therefore a statistically non-significant finding. This might lead to a larger randomized trial to firmly establish the efficacy of the intervention we have studied. A larger trial might also become necessary to more firmly establish moderators of treatment effect as the statistical power to detect these subgroup effects is considerably smaller than the statistical power for the main effect (50).

Finally, it should be noted that we are labeling the intervention as “unguided self-management” because users do not receive regular messages written by clinicians. Mostly, “guided self-management” refers to interventions, where clinicians contact users on a regular basis to enhance engagement with the intervention by giving semi-standardized feedback on the users work with the intervention. A recent RCT compared this type of “guided self-management” with automatically generated standardized messages (51). Both groups only differ with regard to attrition but not with regard to the main outcome—depressive symptoms. In our intervention, users can access a technical support hotline, they will also receive reminders if they have not logged onto the intervention after 5 days and receive automated personalized messages designed to increase their engagement with the intervention and the content covered therein. Therefore, instead of labeling the intervention “unguided” it could also be labeled “with standardized automated guidance.”

In conclusion, the RCT described in this protocol will test the efficacy of the self-management intervention priovi in the treatment of BPD. If this trial is successful, the intervention could eventually become an additional tool for clinicians caring for patients with BPD and may open a new way to increase the availability of evidence-based treatments for patients with BPD.

## Ethics statement

This trial has been approved by the research ethics committee of the University of Lübeck[17-346]. Protocol amendments will be communicated with this committee and posted on ClinicalTrials.gov. All trial participants will provide electronic informed consent before initiating any further study-related procedure. All data will be pseudonymized and the list with the real names and the respective pseudonyms will be stored with password protection. This list will be deleted following the end of data collection and thereafter the data will be anonymous.

## Dissemination policy

Authorship will be granted according to ICMJE criteria (http://www.icmje.org/). Individuals who fulfill authorship criteria will not remain hidden (ghost authors) and will have final authority over manuscript content. The main results of this study will be submitted for publication to a peer-reviewed journal. After this publication, results will also be made available to the general public in lay terminology. Further topics for presentation and publication can be submitted and will be decided by the steering committee. Individual participant data can be shared with researchers who provide a methodologically sound proposal to JK. Proposals may be submitted up to 36 months following publication of the main analysis and will be decided by the steering committee. All patients will consent to data sharing during the informed consent procedure.

## Author contributions

GJ, JK, and US designed the study with substantial input from TB and EF. The steering committee of the study comprises AH, GJ, and JK. JK wrote the manuscript with substantial input from all the authors. All authors commented on the manuscript and approved the final version.

### Conflict of interest statement

Both EF and GJ have received payments for trainings and published books/DVDs on Schema Therapy and treatment of BPD. AH receives payments for a Schema Therapy card set published by Beltz. JK received funding for clinical trials (German Federal Ministry of Health, Servier—distributor of the self-management intervention “Deprexis”), payments for presentations on internet interventions (Servier), payments for workshops and books (Beltz, Elsevier, and Hogrefe) on psychotherapy for chronic depression and on psychiatric emergencies. The remaining authors declare that the research was conducted in the absence of any commercial or financial relationships that could be construed as a potential conflict of interest.
